# The olive knot disease as a model to study the role of interspecies bacterial communities in plant disease

**DOI:** 10.3389/fpls.2015.00434

**Published:** 2015-06-10

**Authors:** Roberto Buonaurio, Chiaraluce Moretti, Daniel Passos da Silva, Chiara Cortese, Cayo Ramos, Vittorio Venturi

**Affiliations:** ^1^Dipartimento di Scienze Agrarie, Alimentari e Ambientali, Università degli Studi di Perugia, Perugia, Italy; ^2^Department of Microbiology, University of Washington, Seattle, WA, USA; ^3^Instituto de Hortofruticultura Subtropical y Mediterránea La Mayora, Universidad de Málaga-Consejo Superior de Investigaciones Científicas, Málaga, Spain; ^4^Bacteriology Group, International Centre for Genetic Engineering and Biotechnology, Trieste, Italy

**Keywords:** olive knot disease, *Pseudomonas syringae*, *Pantoea agglomerans*, *Erwinia toletana*, *Erwinia oleae*, microbiome, biofilm, plant endophytes

## Abstract

There is an increasing interest in studying interspecies bacterial interactions in diseases of animals and plants as it is believed that the great majority of bacteria found in nature live in complex communities. Plant pathologists have thus far mainly focused on studies involving single species or on their interactions with antagonistic competitors. A bacterial disease used as model to study multispecies interactions is the olive knot disease, caused by *Pseudomonas savastanoi* pv. savastanoi (*Psv*). Knots caused by *Psv* in branches and other aerial parts of the olive trees are an ideal niche not only for the pathogen but also for many other plant-associated bacterial species, mainly belonging to the genera *Pantoea*, *Pectobacterium*, *Erwinia*, and *Curtobacterium*. The non-pathogenic bacterial species *Erwinia toletana*, *Pantoea agglomerans*, and *Erwinia oleae*, which are frequently isolated inside the olive knots, cooperate with *Psv* in modulating the disease severity. Co-inoculations of these species with *Psv* result in bigger knots and better bacterial colonization when compared to single inoculations. Moreover, harmless bacteria co-localize with the pathogen inside the knots, indicating the formation of stable bacterial consortia that may facilitate the exchange of quorum sensing signals and metabolites. Here we discuss the possible role of bacterial communities in the establishment and development of olive knot disease, which we believe could be taking place in many other bacterial plant diseases.

## Introduction

In natural environments, bacterial species are rarely found alone and most often live in communities where they commonly form multispecies biofilms, which are composed of bacterial cells attached to a surface and to each other and are embedded in a self-produced matrix of extracellular polymeric substances (De Beer and Stoodley, 2006). Bacteria in biofilms are sessile and are well protected against various stresses including host defenses; this protective effect can be further enhanced in a synergistic manner in a multispecies biofilm in comparison to a monospecies one (Jefferson, 2004; Burmølle et al., 2006, 2014). The complex microbial community present in the human oral cavity, which contains more than 700 microbial species (mainly bacteria), is one of the best-described and has become the paradigm of multispecies biofilms (Kolenbrander et al., 2010; Guo et al., 2014). In this system, individual bacterial species compete and collaborate with other neighboring ones through metabolic interactions, which not only modify the local microenvironment such as pH and the amount of oxygen, making it more suitable for the growth of other species, but also provide a metabolic framework for the participating microorganisms by maximizing their potential to extract energy from limited substrates (Kolenbrander et al., 2010; Guo et al., 2014). However, the interplay between microbial species and the host in oral cavity can be seen as a fragile equilibrium, and disruption of this balance can have detrimental effects on health. Dental carries and periodontitis are just a few examples of polymicrobial oral diseases (Ramsey et al., 2011; Demuyser et al., 2014).

Multispecies infections have also been well described in chronic mammalian infections, such as the ones occurring in the lungs of cystic fibrosis patients (Burmølle et al., 2010). Multispecies biofilms also occur in bacteria residing in non-host environments including soil, seawater, and artificial habitats such as drinking water distribution systems, boat hulls, and dairy production processes (Stewart, 2002; Burmølle et al., 2006; Hangler et al., 2009; Kolenbrander et al., 2010; Schwering et al., 2013).

Olive knot disease caused by *Pseudomonas savastanoi* pv. *savastanoi* (hereafter Psv) is considered one of the most serious diseases affecting olive trees (*Olea europaea* L.) in most olive growing regions worldwide and mainly in Mediterranean countries, which can lead to severe damage in olive groves, causing serious losses in terms of production (Quesada et al., 2012). Recent studies have shown that the tumors (knots) caused in olive trees by this bacterium contain a multispecies bacterial community and that bacterial species of this microbiome collaborate with the pathogen in increasing the disease severity (Hosni et al., 2011; Passos da Silva et al., 2014). Recent sequencing of the genome of *Psv* strains isolated in France (Rodríguez-Palenzuela et al., 2010; Bardaji et al., 2011) or Italy (Moretti et al., 2014c) as well as sequencing of the genome of three non-pathogenic bacterial species frequently associated with the olive-knot microbiome, namely *Pantoea agglomerans* (hereafter *Pa*; Moretti et al., 2014d), *Erwinia toletana* (hereafter *Et*; Passos da Silva et al., 2013), and *Erwinia oleae* (hereafter *Eo*; Moretti et al., 2014b), have shed light on possible interspecies interactions making this niche a model to study the establishment of a multispecies community in a plant disease.

In this review, after briefly introducing the olive knot disease, we focus on the olive-knot microbiome, reporting the current knowledge on the role of the harmless/beneficial endophytes *Pa*, *Et*, and *Eo* in disease development. Finally, we discuss possible future directions in the study of this bacterial multispecies interaction and their role in the disease.

## The Olive Knot Disease

The olive knot disease, caused by the Gram-negative phytopathogenic bacterium *Psv*, is characterized by the overgrowth formation (tumors, galls, or knots) in the aerial part of the olive trees, mainly on stems and branches and, occasionally, on leaves and fruits (Iacobellis, 2001; Young, 2004; Quesada et al., 2012; Ramos et al., 2012). It can be considered a chronic disease as its symptoms persist and recur for many years in olive trees. *Psv* is able to survive as an epiphyte on olive phyllospere (Ercolani, 1971; Quesada et al., 2010), where its population is subjected to seasonal fluctuation: higher in spring and fall than in winter and summer (Ercolani, 1971, 1978; Varvaro and Surico, 1978; Quesada et al., 2007). Wounds caused by harvesting, pruning, hail, frost, and leaf scars, permit *Psv* to enter in the plant tissues and to generate knots. Analogously to the tumor-inducing bacterium, *Agrobacterium tumefaciens* (Sheng and Citovsky, 1996), *Psv* may need plant-released signals from the wounds to activate the tumor formation, as when it enters through stomata it is not able to generate any visible symptoms (Surico, 1993). In fresh wounds of olive trees, the pathogen initially colonizes the tissues around the infection point and, through pectolytic and hemicellulolytic enzymes, disrupts the integrity of the host cells, producing cavities that are filled with the bacterium; alternatively, it can directly invade the xylem vessels (Marchi et al., 2009; Rodríguez-Moreno et al., 2009; Maldonado-González et al., 2013). Successively, bacterial virulence factors, mainly indol-3-acetic acid (IAA) and cytokinins, provoke an increase in plant cell size (hypertrophy) followed by an abnormal cell division (hyperplasia; Rodríguez-Moreno et al., 2008; Quesada et al., 2012). It is worth noting that bacterial IAA is synthesized from tryptophan in a pathway different from those present in plants, in which the genes *iaaM* (tryptophan monooxygenase) and *iaaH* (indoleacetamide hydrolase) are involved (Surico and Iacobellis, 1992; Aragón et al., 2014). Besides the phytohormones, which play a pivotal role, other virulence factors are involved in disease development. Analogously to many Gram-negative phytopathogenic bacteria, *Psv* also expresses its pathogenicity/virulence through the production of a type III secretion system (T3SS), by which it injects into plant cells more than 30 effectors involved in its virulence (Pérez-Martínez et al., 2010; Ramos et al., 2012; Matas et al., 2014). T3SS mutants were not able to multiply in olive tissues and induce the formation of knots in woody olive plants (Sisto et al., 2004; Pérez-Martínez et al., 2010; Ramos et al., 2012). However, when young micropropagated olive plants were inoculated with T3SS mutants, tumors were generated without the formation of necrosis and internal open cavities, which are generated by the wild-type strain (Pérez-Martínez et al., 2010). A recent signature-tagged mutagenesis survey of the Psv NCPPB 3335 genes required for full fitness in olive plants identified a total of 58 genes, most of which are required for the expression of the highest disease severity in woody olive plants. Metabolic-related genes disrupted in these strains included genes encoding enzymes involved in the biosynthesis of nine amino acids (arginine, glutamic acid, histidine, isoleucine, leucine, methionine, proline, tryptophan, and valine) and three vitamins (biotin, cobalamin, and thiamine), as well as genes encoding putative sulfate, citrate, and amino acid transporters. Furthermore, this study unravels novel factors involved in the virulence of this pathogen, such as the Sec pathway, the type IV secretion system, a suite of genes involved in detoxification or tolerance to reactive oxygen species (ROS), peptidoglycan-related genes and factors involved in the metabolism of cyclic di-GMP (c-di-GMP; Matas et al., 2012). Quorum sensing (QS) intercellular regulation and communication system, mediated by *N*-acyl homoserine lactones (AHLs) also plays a major role in *Psv* virulence (Hosni et al., 2011). AHLs are produced by an AHL synthase belonging to the LuxI-protein family, while a transcriptional sensor/regulator, belonging to the LuxR family, then forms a complex with the cognate AHL at threshold (“quorum”) concentrations thereby affecting the transcription of target genes (Fuqua et al., 2001). It has been demonstrated that knockout mutants of the *luxR* and *luxI* homolog genes of *Psv* showed a reduced virulence when inoculated in olive plants (Hosni et al., 2011).

## Olive-Knot Microbiome

The presence inside the olive knots of bacterial species other than *Psv* has been documented for over one century. Savastano (1886) isolated from olive knots a yellow-pigmented bacterium that was initially wrongly considered the causal agent of the disease. The error was highlighted by Petri (1907), who, on the basis of Savastano’s description, identified the yellow bacterium as *Ascobacterium luteum* [ = *Bacterium* (*Erwinia*) *herbicola*, now *Pa* (Gavini et al., 1989)]. Savastano was able to fulfill Koch’s postulates as he used a mixed inoculum containing prevalently *Psv* (Marchi et al., 2006). Besides *Pa*, which has often been reported to be associated with olive knots (Surico and Lavermicocca, 1989; Fernandes and Marcelo, 2002; Ouzari et al., 2008; Hosni et al., 2011), other endophytes have been reported to live inside this ecological niche, such as the characterized species *Et* (Rojas et al., 2004) and *Eo* (Moretti et al., 2011). According to Hallmann et al. (1997), we use hereafter the term endophyte to indicate any bacterium that can be isolated from surface-disinfected plant tissue or extracted from inside the plant (i.e., inside the olive knots), and that does not visibly harm the plant.

A culture independent metagenomic approach, carried out in knot samples coming from different Italian regions and based on the amplification and sequencing of the hypervariable 16S rRNA regions, revealed that the gammaproteobacteria class was by far the most represented, accounting for up to 90% of the total bacterial population, with the most commonly found orders Pseudomonadales (mainly *Psv* which was estimated to represent approximately 50% of the total bacterial load in the knot) and Enterobacteriales (Passos da Silva et al., 2014). Within the Enterobacteriales, the most abundant bacteria belong to the *Pantoea* genera, while a common core of other bacterial genera was evident composed of *Clavibacter*, *Curtobacterium*, *Enterobacter*, *Erwinia*, *Hymenobacter*, *Kineococcus*, *Pectobacterium*, and *Sphingomonas* (Passos da Silva et al., 2014).

## Current Knowledge on the Role of the Endophytes

Among the endophytes living in the olive knots, the bacterial species *Pa*, is the most investigated. It is widespread in many diverse natural and agricultural habitats and is associated with many plants as a common epiphyte and endophyte (Kobayashi and Palumbo, 2000; Lindow and Brandl, 2003). Adaptation of *Pa* to diverse microenvironments most probably allowed a rapid evolution even as a plant pathogen inducing gall formation on gypsophila (Cooksey, 1986), beet (Burr et al., 1991), Douglas fir (DeYoung et al., 1998), wisteria (Opgenorth et al., 1994), and cranberry (Vasanthakumar and McManus, 2004). The pathogenicity of *Pa* on gypsophila and beet plants, the strains belonging to the pvs. *gypsophilae* and *betae* respectively, is due to the presence in their genomes of plasmids presumably acquired through horizontal gene transfer (HGT; Manulis and Barash, 2003). The pathogenicity islands present in these plasmids mainly harbor the *hrp* gene cluster encoding type III effector proteins and enzymes for IAA and cytokinin biosynthesis (Manulis and Barash, 2003; Barash and Manulis-Sasson, 2009). *Pa* may be also involved indirectly in pathogenesis, by modifying the predisposition of plants to infection and/or by modifying the virulence and the activity of the true phytopathogen (Gibbins, 1978). The fact that the isolated colonies of *Pa* from olive knots were in average 15 times more numerous than those of *Psv* prompted Fernandes and Marcelo (2002) to investigate on the possible interaction between the two bacterial species. They observed that knots induced by *Psv* alone were smaller than those obtained in olive plants co-inoculated with *Psv* and *Pa*. Similar results were obtained by Marchi et al. (2006) and Hosni (2010). *Pa* strains isolated from olive knots are not pathogenic on olive trees, though the majority are able to induce hypersensitive reaction (HR) in tobacco leaves (Marchi et al., 2006; Hosni, 2010), a characteristic shared by many phytopathogenic bacteria (Buonaurio, 2008). The recent genome sequencing of the HR-inducing strain DAPP-PG 734 of *Pa*, isolated from an olive knot (Moretti et al., 2014d), revealed the presence of a complete *hrc*/*hrp* gene cluster, the sequences having high similarity with those of the pear pathogens, *Erwinia amylovora* and *Erwinia pyrifoliae* (Moretti et al., 2014a). On the basis of this information, its pathogenicity on immature and mature pear fruits was evaluated (Moretti et al., 2014a). This strain induced a weak browning only on mature pear fruits (Moretti et al., 2014a). The production of IAA by *Pa* strains, that in some cases reaches values similar to those produced by *Psv* (Marchi et al., 2006; Hosni, 2010), is one of the hypotheses explaining the higher knot size observed in the co-inoculation experiments.

*Et* and *Eo* are also IAA-producing olive knot endophytes, which also caused an increase in disease severity when co-inoculated with *Psv* in olive plants (Hosni, 2010; Hosni et al., 2011). Interestingly, the growth in olive plants of *Pa*, *Et*, and *Eo* increases significantly in the presence of *Psv* (Marchi et al., 2006; Hosni, 2010). Recently the genomes of one strain of *Et* and one of *Eo* have been determined (Passos da Silva et al., 2013; Moretti et al., 2014b); analysis of these genomes might provide clues to their life inside the olive knot.

**FIGURE 1:**
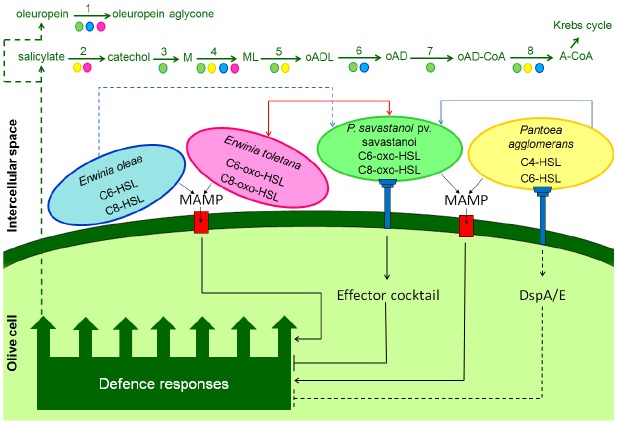
**Model representing the interactions between olive cells, *Pseudomonas savastanoi* pv. *savastanoi* (*Psv*, green bacterial cell) and the endophytes, *Pantoea agglomerans* (*Pa*, yellow), *Erwinia toletana* (*Et*, pink), and *Erwinia oleae* (*Eo*, blue).** In the intercellular space of the olive tissues, *Psv* and *Et* produce and perceive the quorum sensing signals C6-3-oxo-HSL and C6-3-oxo-HSL (symmetrical sharing, red line). *Pa* (blue line), and probably *Eo* (dashed blue line), establish an asymmetrical sharing with *Psv*. In fact, *Pa* produce the quorum sensing signals C4-HSL and C6-HSL, which one or both are perceived by *Psv*; *Eo* produces C6-HSL and C8-HSL. Microbial associated molecular patterns (MAMPs) are produced by *Psv* and the other endophytes (e.g., flagellin); they are recognized by specific plant receptors (red box) and defense responses are activated in the host plant (MAMP-triggered immunity, MTI). Among the defense responses, accumulations of salicylic acid (SA) and other phenolic compounds (e.g., oleuropein) can occur in the intercellular space. By contrast, the following enzymes present in the genomes of *Psv*, *Pa*, *Et*, and *Eo* could collaborate in demolishing SA and oleuropein: (1) β-glucosidase (EC 3.2.1.21); (2) salicylate hydroxylase (EC 1.14.13.1); (3) catechol 1,2-dioxygenase 1 (EC 1.13.11.1); (4) muconate cycloisomerase (EC 5.5.1.1); (5) muconolactone isomerase (EC 5.3.3.4); (6) β-ketoadipate enol-lactone hydrolase (EC 3.1.1.24); (7) 3-oxoadipate CoA-transferase subunit A (EC 2.8.3.6); and (8) acetyl-CoA C-acyltransferase (EC 2.3.1.16). In addition, through the type III secretion system (pilus Hrp), *Psv* injects a cocktail of effectors inside the olive cells, many of which are involved in suppressing plant defense responses. Also *Pa*, which has a complete *hrc/hrp* gene cluster, could inject the defense-suppressive effector DspA/E. M = *cis*, *cis*-muconate; ML, (+)-muconolactone; oADL, 3-oxoadipate-enol-lactone; oAD, 3-oxoadipate; oAD-CoA, 3-oxoadipyl-CoA; A-CoA, acetyl-CoA.

Members of multispecies bacterial communities communicate and cooperate via the exchange of public goods and chemical signaling molecules, while also competing for space and resources (Kerényi et al., 2013; Venturi and Fuqua, 2013). Accumulating evidence suggests that *Et*, *Pa*, and *E*o form stable interspecies community with *Psv* and they communicate through QS N-AHL signals. *Et* as well as *Psv* produce C6-3-oxo-HSLs and C8-3-oxo-HSLs, *Pa* produces C4-HSLs and C6-HSLs whereas *Eo* produces C6-HSLs and C8-HSLs (Hosni, 2010; Hosni et al., 2011). The fact that *Et* and *Psv* produce structurally the same AHLs, prompted Hosni et al. (2011) to investigate whether they were able to share these signals. Co-inoculation experiments between a *Psv* AHL synthase mutant, which is unable to produce AHLs and was less virulent, and the wild-type strain of *Et* revealed that the *Psv* mutant was able to induce knot formation in olive plants just like the wild-type strain. It is reasonable to hypothesize that the two strains form a consortium, where *Et* rescues the virulence of *Psv* by providing AHLs to the *Psv* mutant, which is then able to induce AHL QS target gene expression resulting in olive knot formation. Similar pairwise inoculations were also performed with a wild-type strain of *Pa* and the *Psv* AHL synthase mutant and only partial restoration of knot formation was observed. This could be due to *Pa* producing structurally close but different AHLs than *Psv* (Hosni et al., 2011). Although analogous experiments with *Eo* have not yet been performed, it is possible to assume that *Eo* has a behavior similar to *Pa*, considering that both produce C6-HSLs. It is believed that in the wild, it is not uncommon to share AHL signals bacteria also due to the promiscuity of the LuxR regulator-receptor protein, which are often capable of responding to structurally different AHL signals (Subramoni and Venturi, 2009; Coutinho et al., 2013). On the basis of the above described results and the model proposed by Kerényi et al. (2013), it is possible to schematize at least two possible relationships (symmetrical and asymmetrical) between the “niche-maker” *Psv* and the other investigated olive-knot “resident” endophytes, *Et*, *Pa*, and *Eo*, all of which produce IAA and can therefore be considered a public good within the consortium (Figure 1). *Psv* and *Et* can mutually utilize AHL signals and public goods (symmetrical relationship). An asymmetrical relationship on the other hand can occur between *Psv* and *Pa* or *Eo*; *Psv* possibly exploits the signals of *Pa* and *Eo* and vice versa.

The hypothesis of formation of a stable consortium between *Psv* and the harmless resident of the olive knots, *Et*, *Pa*, or *Eo* is further strengthened by the epifluorescence microscopy experiments. Using strains of *Et*, *Pa*, *Eo*, and *Psv* expressing autofluorescent proteins, it has been demonstrated that *Psv* intimately co-localizes with *Et*, *Pa*, and *Eo* (Cortese et al., 2014; Passos da Silva et al., 2014).

## Possible Role of the Resident Microbiota

In this section, based on scientific literature as well as *in silico* bioinformatics analysis, we address the following questions in order to begin to better understand the role of the resident microbiota, *Et*, *Eo*, and *Pa*, in the olive knot disease development.

(1) Does the resident microbiota possess the genetic traits characterizing an endophytic life style?
(2) Can resident microbiota produce biofilms?
(3) Do resident microbiota suppress plant defense responses?

### Endophytic Lifestyle

Endophytic bacteria colonize inner host tissues without damaging the plant host or eliciting strong defense responses. The molecular basis of this interaction is currently generally unknown. In addition, it is not clear whether these interactions are shared/common among different endophytes; however, there is evidence that several endophytic bacteria result in an array of beneficial effects for the plant (Ryan et al., 2008; Reinhold-Hurek and Hurek, 2011). Evidence of a rich and diverse endophytic bacterial population in several plant hosts has been determined by culture independent omic methodologies (Lundberg et al., 2012; Sessitsch et al., 2012). Currently we have very little knowledge on how bacteria adapt, colonize, and live as endophytes in plants.

Endophytes colonize the intercellular spaces in the epidermal and cortical regions and in some cases, at lower cell densities, can also invade the vascular tissue therefore allowing the spreading into shoots. Most endophytes are believed to enter inside the plant at the roots via the rhizosphere; this relies in part on motility, root penetration, and adaptation to the plant intercellular environment (Compant et al., 2010; Reinhold-Hurek and Hurek, 2011). This adaptation to a new environment also requires that the endophyte overcomes plant defense responses and the whole process can be viewed as similar to the one encountered by incoming phytopathogens with the major difference being that for endophytes, the plant–bacteria interaction is mutualistic. This is an important open question, i.e., how can endophytes colonize in high numbers a plant tissue without eliciting significant plant response and plant damage? Because they are able to colonize a niche similar to that of plant pathogens, they can become possible biocontrol agents to control incoming phytopathogens. Future studies will need to clarify the molecular mechanisms of adaptation, recognition, and communication between harmless or beneficial endophytes and the plant. In addition, which specific features belong to endophytes in comparison to soil, rhizosphere, and pathogenic microorganism?

In order to obtain insights into strategies of endophytic lifestyle, scientists are beginning to sequence the genomes of several endophytes and perform comparative genome studies. The initial bioinformatic analyses have not yet deduced genetic traits characterizing an endophyte (Mitter et al., 2013). A wide spectrum of traits necessary for endophytic lifestyle is likely to exist including motility, chemotaxis, adhesion, signaling, membrane transport, secretion, and degradation of plant polymers and organic compounds as a recent study on an endophyte response at the molecular level to the plant environment has recently evidenced (Reinhold-Hurek and Hurek, 2011; Coutinho et al., 2015). As more genomes of endophytes are sequenced as well as genetic and molecular studies performed, it is expected that the main traits necessary for endophytic colonization will be revealed. This research will probably pick up quickly as it is believed that endophytes, as well as other plant-associated bacteria (collectively called plant microbiome), contribute significantly to plant health.

Similarly to *Psv*, it is possible that many of the bacteria living as endophytes in olive knots are also resident in the olive phyllosphere and enter in the plant tissues mainly through the wounds and not via the rhizosphere. In fact Ercolani (1978) isolated many bacterial species from olive phylloplane and *Pa* is one of the most represented. It cannot be excluded however that a part of the olive-knot endophytic residents originate from the rhizosphere of plant roots. In this sense and related with olive knot disease, *Pseudomonas fluorescens* strain PICF7, a native endophyte of olive roots and an effective biocontrol agent against the soil-borne fungus *Verticillium dahliae* (Mercado-Blanco et al., 2004), has been reported to establish and persist in olive stem tissues upon artificial inoculation. Although this strain is not able to suppress olive knot disease, its presence decreases pathogen population size and confine *Psv* at inner regions of the tumors (Maldonado-González et al., 2013). Genome analysis of this strain recently revealed genes that might be related to biocontrol and endophytic traits, such as encoding for secretion systems, siderophores, detoxifying compounds, or volatile components (Martínez-García et al., 2015). Moreover, and as mentioned above, a recent metagenomic study on microbiome of olive knot indicated the presence of approximately 50% of the bacterial load being *Psv* and the remaining 50% being many different bacterial species belonging to different genera present in the olive knot (Passos da Silva et al., 2014). Many of the bacterial genera are also present in the rhizosphere thus it is likely that they are endophytes entering the olive plant via the roots. The rich bacterial endophytic population in the olive knot is likely to involve several mechanisms of molecular interaction including QS signal sharing and metabolic complementarity. This niche is therefore an excellent model to study all the different interactions taking place among an endophytic community, particularly among harmless bacteria as well as harmless bacteria and *Psv*.

### Biofilm Production

Successful plant colonization is often linked to the ability of bacteria to form adherent microbial populations (Danhorn and Fuqua, 2007). This sessile form of bacterial life, also called biofilm, is extensively studied and very complex. Bacterial attachment to plant surfaces involves several proteins including adhesins, amyloid curli fimbriae, type I fimbriae, type IV pili, and flagella (Jefferson, 2004; Barken et al., 2008; Bahar et al., 2009; Wang et al., 2013; Heindl et al., 2014; Yaron and Römling, 2014) as well as production of exopolysaccharides (EPS), capsular polysaccharides (CPS), and extracellular DNA (eDNA). Among the adhesins, filamentous hemagglutinin-like (FHA) proteins play a fundamental role in plant–bacteria interactions. FHA proteins of *Xanthomonas axonopodis* pv. *citri* is required for tissue colonization being mainly involved in surface attachment and biofilm formation (Gottig et al., 2009). In *Pseudomonas* on the other hand, CdrA (an FHA-like protein) was shown to be associated with the Psl polysaccharide, which is a biofilm component (Borlee et al., 2010). Since FHA genes are present in a number of bacterial endophytes, it has been hypothesized that they play a crucial role in the invasion of plant tissues and in biofilm formation (Mitter et al., 2013). BlastP and analysis of neighboring genes demonstrated that *Eo* and *Et* possess FHA-like genes that could aid the formation of a stable biofilm structure. Further *in silico* analysis and experimental studies could demonstrate the importance of these high molecular weight proteins in biofilm formation as well as bacteria–bacteria and plant–bacteria interactions.

*Psv* strains are able to form air–liquid biofilms over abiotic surfaces (Ude et al., 2006; Pérez-Mendoza et al., 2014). Furthermore, visualization of olive knot sections by scanning confocal laser microscopy (SCLM) and electron microscopy has allowed visualization of the organization of *Psv* cell clusters (microcolonies and biofilms) inside the host tissues. In fact, *Psv* is visualized inside olive knots forming biofilms composed of a multilayer of bacterial cells colonizing the surface and interior of plasmolysed host cells (Rodríguez-Moreno et al., 2009). Moreover, calcofluor epifluorescent microscopy has identified cellulose as the EPS component in biofilms produced by *Psv* strains (Ude et al., 2006). Thus, cellulose production by *Psv* cells might play a relevant role both in the virulence of *Psv* and in the establishment of mixed-species community biofilms (*Psv*, *Pa*, *Et*, and *Eo*) inside olive knots. In relation to virulence determinants, the size of the knots induced by *Psv* in olive plants has been shown to be dependent on the activity of several enzymes involved in the metabolism of the second messenger c-di-GMP, which in turn, regulate EPS production and biofilm formation (Pérez-Mendoza et al., 2014; Aragón et al., 2015). Further studies addressing the composition of the EPS matrix surrounding mixed-species bacterial biofilms established inside olive knots would be necessary to shed light in this hypothesis. Microscopic visualization of olive knots induced on *in vitro* micropropagated olive plants after mixed inoculation of *Et* and *Psv* revealed that the distribution of *Et* cells closely matched the position of *Psv* cells (Passos da Silva et al., 2014). Preliminary and similar results were obtained by Cortese et al. (2014) when *Eo* and *Pa* were assayed instead of *Et*. This close proximity possibly suggests that mixed biofilms occur inside olive knots; however, this hypothesis needs to be experimentally tested.

It was recently discovered that one mechanism by which Gram-negative bacteria that are in close proximity to each other can interact is by contact-dependent transport of effector proteins from a donor cell to a recipient cell via the activity of an apparatus known as the type VI secretion system (T6SS; Russell et al., 2014). Initially found to deliver effector proteins into eukaryotic cells, T6SS seems mainly involved in interbacterial interactions. Killing of bacteria (non-cooperators) that weaken the stability of a bacterial community and the contribution to maintain the three-dimensional architecture of bacterial communities is one of the potential roles of T6SS (Russell et al., 2014). Interestingly, *Eo* and *Pa* genomes harbor regions of 6.3 and 7.1 kb, respectively, flanked by mobile element proteins, which contain genes belonging to the T6SS, namely the loci *vgrG* and *rhs* which encode for valine/glycine-repeat protein G (Chang et al., 2014; Hachani et al., 2014) and rearrangement HotSpot elements (Koskiniemi et al., 2013), respectively. The presence of the flanking mobile elements, the higher G + C content of this region with respect to the G + C content of the whole genomes of *Pa* and *Eo*, and the high similarity (99%) with *Psv* homolog genes, suggests an acquisition of this region via HGT possibly from *Psv*. We can hypothesize that strains of *Pa* and *Eo* have evolved together with *Psv* and that they collaborate with *Psv* in the maintenance the stability of the olive-knot consortium through T6SS. In addition, the biofilms, which are probably formed by multispecies communities in the olive knots provide an excellent environment for DNA exchange via HGT since cells are in close proximity for a long time and DNA can be trapped within the extracellular matrix (Madsen et al., 2012). It is therefore tempting to speculate that these harmless residents in the knot can in the future evolve to become pathogenic by acquiring the loci from neighboring pathogenic cells.

### Suppression of Plant Defenses

Plants are exposed to a vast number of potential pathogens, against which they try to defend themselves through a multilayered system of preformed and induced defenses, both structural and biochemical. Few investigations have been carried out on the defense responses of olive plants against *Psv*, mostly because resistant olive cultivars to olive knot are not available (Penyalver et al., 2006). Important information on olive defense responses are provided by the studies on the resistance induced by the biocontrol agent *P. fluorescens* PICF7 (Schilirò et al., 2012; Gomez-Lama Cabanas et al., 2014). Unfortunately, this resistance is effective against Verticillium wilt (Prieto et al., 2009; Schilirò et al., 2012) but not on olive knot disease (Maldonado-González et al., 2013). Thus, defense responses against *Psv* are essentially limited to studying weakly resistant or susceptible olive plants to *Psv* infections.

Among the structural induced defenses, Temsah et al. (2008) observed lignin deposits on cell walls of parenchyma olive cells around cavities and injured tissues caused by *Psv*. They also reported that new periderm was produced at the surface of the olive knots, which constitutes a barrier impairing the entrance of other pathogens and saprophytes.

Regarding the biochemical induced defenses, a number of histological and biochemical studies reported a marked accumulation of phenolic compounds in olive knots (Roussos et al., 2002; Cayuela et al., 2006; Marchi et al., 2009), a resistance mechanism well documented in many other bacterial diseases (Buonaurio, 2008). More precisely the following two compounds are involved, the secoiridoids oleuropein and verbascoside. The phenolic glucoside oleuropein, which is an ester of elenolic acid with hydroxytyrosol linked to glucose through a 1,4-beta bond, showed its toxicity against *Psv* in the 0.1–1 mM concentration range, while oleuropein aglycone and hydroxytyrosol in the ranges 0.1–10 and 1–10 mM, respectively (Capasso et al., 1995). Verbascoside is a heterosidic ester of caffeic acid and hydroxytyrosol linked to rhamnose and glucose, which has antimicrobial activity (Pardo et al., 1993), though not documented for *Psv*. Since phenolic compounds accumulate inside olive cells, especially in the vacuoles (Uccella, 2001), we can assume their antibacterial activity is manifested when plant cell disruption occurs. Therefore it is reasonable to speculate that this hypothetical defense response is effective during the early stage of the infection process, when *Psv* induces cavity formation in the plant tissues through pectolytic and hemicellulolytic enzymes, as well as during the later stages, when large cavities formed after the collapse of intercellular plant cells. By contrast, *Psv* and the endophytes discussed here may provoke the degradation of phenolic glucoside compounds through enzymatic activities. Genes for beta-glucosidases are present in *Psv*, *Et*, and *Eo*. These enzymes may cleave the glucoside moieties and allow the molecule to be further mineralized.

The phytohormone salicylic acid (SA) could also be involved in the defense against *Psv*. Besides acting as a signaling molecule in plant defense against biotrophic and hemibiotrophic plant pathogens (Boatwright and Pajerowska-Mukhtar, 2013), SA may function as an antimicrobial agent. Cameron and Zaton (2004) reported an accumulation of SA in the intercellular space of old *Arabidopsis* plants and correlated it with the age-related resistance to *Pseudomonas syringae* pv. *tomato* DC 3000. In fact, they also demonstrated that SA has antimicrobial activity against the bacterium and that the destruction of SA in the intercellular space by salicylate hydroxylase reduced the resistance level in Arabidopsis plants. If SA builds up in the olive knot apoplast it could result in antimicrobial activity against *Psv* (which is likely since *Psv* cannot convert SA to catechol as evidenced by Passos da Silva et al., 2014); this can be potentially alleviated by *Et* and *Pa* since these bacteria possess the salicylate hydroxylases gene, which converts SA to catechol. Catechol is one of the most important central intermediates in the aerobic bacterial metabolism of aromatic compounds (Harwood and Parales, 1996) and it is toxic to *Psv* (Capasso et al., 1997). This phenol may be further degraded and detoxified by the collaborative action of *Psv*, *Et*, *Eo*, and *Pa* enzymes by *ortho* cleavage via the 3-oxoadipate pathway, which lead in to the Krebs cycle (Harwood and Parales, 1996; Figure 1). This is a possible example of how harmless endophyte residents can aid the growth of *Psv* pathogen via metabolic complementarity. Interestingly, in the genomes of several *Psv* strains there is a region of about 15 kb, named VR8 which is absent in all sequenced *P*. *syringae* strains infecting herbaceous plants. This region is however present in *P. syringae* pathovars infecting woody hosts, such as *aesculi*, *morsprunorum*, and *actinidiae* (Ramos et al., 2012; Moretti et al., 2014c). Among other genes encoded in this region there is also the *catBCA* operon which is involved in the degradation of the catechol possibly allowing a selective advantage for growth in woody hosts.

There is a growing body of evidence that the T3Es injected inside plant cells by phytopathogenic bacteria suppress host defenses, i.e., PAMP-triggered immunity (PTI) and effector-triggered immunity (ETI; Jones and Dangl, 2006). This occurs via different routes such as interference with immune receptor signaling, blocking of RNA pathways, and vesicle trafficking and alteration of organelle function (Block and Alfano, 2011). Matas et al. (2014) demonstrated that seven *Psv* T3Es (HopA1, AvrRpm2, HopAA1, HopAZ1, HopBK1, HopBL1, and HopBL2) suppress the PTI and two of them (HopAZ1 and HopBL1) also suppress ETI. We cannot exclude that T3Es are produced by some endophytic bacteria of the olive-knot microbiome, which collaborate with *Psv* in mitigating host defenses. In fact, the HR-inducing strain of *Pa*, isolated from the olive microbiome and characterized by Moretti et al. (2014d), harbors the *dspA/E* and *dspB/F* genes adjacent to its complete *hrc*/*hrp* gene cluster, which encode for the homologs *Erwinia amylovora* effector DspA/E and its chaperone DspB/F, which in turn is essential for DspA/E secretion and stability (Gaudriault et al., 2002; Boureau et al., 2006). Among the *E. amylovora* T3Es, DspA/E plays a pivotal role in disease development as mutants defective for this effector are non-pathogenic and are unable to grow in host plants (Barny et al., 1990; Gaudriault et al., 1997) and non-host tobacco (Oh et al., 2007). DspA/E belong to the AvrE effector superfamily, which is widespread among type III-dependent phytopathogenic bacteria, playing a crucial role during bacterial pathogenesis by suppressing PTI and are also present in the genome of non-pathogenic plant associated bacteria (Degrave et al., 2013). AvrE-T3Es inhibit SA-mediated plant defenses, interfere with vesicular trafficking and promote bacterial growth *in planta*. We can hypothesize that HR-inducing strains of *Pa* are able to deliver the effector DspA/E in olive cells, which, in turn, suppresses the host defenses and therefore facilitate the growth of microbiota inside the olive-knot (Figure 1). Interestingly, *Eo* genome harbors the *dspA/E* gene encoding proteins showing very high identity (99%) with those of *Pa*, suggesting a possible evolution in the olive-knot niche. However, differently from *Pa*, *Eo* does not possess a T3SS, therefore we do not know if it is able to secrete DspA/E effector.

### Future directions and Concluding Remarks

It is now evident that a variety of bacterial species coexist in the natural environment building a network of interactions; this has led to the emergence of a new discipline which has been coined as sociomicrobiology (Parsek and Greenberg, 2005). The most common form of social behavior in bacteria is the production of public goods (e.g., siderophores, extracellular enzymes, secondary metabolites), which benefit all individuals of the community, both cooperators, and defectors or cheaters (West et al., 2006). From an evolutionary point of view, it is known that microbial biodiversity is an important driver of ecological processes and the evolutionary history predicts the stability of cooperation in multispecies bacterial communities (Jousset et al., 2013). The complex social behaviors of bacterial communities have been extensively investigated in *Pseudomonas aeruginosa* as the main actor, now considered a reference model to study fundamental questions about the costs and benefits of cooperation. These include the selective pressures that lead to cooperative behavior and the advantages of controlling cooperative behaviors by communication (for more detailed information, see De Vargas Roditi et al., 2013; Mitri and Foster, 2013; Tashiro et al., 2013). In the olive knot model of multispecies bacterial community, it appears that *Psv* is a social bacterium, which has not evolved to cause disease independently or to live alone inside the olive knot, in spite of its capability to induce alone knot formation in olive trees. In fact, as documented in a number of olive-growing countries and in different and distant locations of the same country, bacterial endophytes, mainly belonging to *Pantoea* and *Erwinia* genera, are constantly associated with *Psv* inside olive knots (Fernandes and Marcelo, 2002; Ouzari et al., 2008; Hosni et al., 2011; Passos da Silva et al., 2014). In addition, the presence of endophytes in the knots is not only observed in nature but also in plants artificially inoculated with *Psv*. Therefore, Psv is likely to find benefit in living in a multispecies community, possibly because it can better exploit plant nutrients as well as contrasting plant defenses.

Several possible contributions to virulence by the resident olive-knot bacteria have already been mentioned in the previously sections including production of phytohormones, biofilm formation, degradation of plant phenolic compounds, c-di-GMP signaling, production of T3 effectors, production of secreted cellulolytic enzymes, and QS signal sharing. An important aspect which merits attention in addition to the possible contribution of virulence is that, resident bacteria could be involved in the formation of a stable multispecies community which allows the pathogen and other bacteria to be most fit to attack plant cells and consequently grow in the olive-knot. This stability therefore needs to avoid diverse growth incompatibilities such as contact dependent mechanisms (Blango and Mulvey, 2009), production of antibacterial compounds and nutrient competition. Common co-isolation of bacterial species from a given niche does not automatically imply cooperation or synergism between them, but is an indication that some type of interaction is taking place. Importantly further studies are needed to investigate these interactions, for example: (i) *in vitro* analysis can focus on growth conditions trying to mimic what occurs in the olive knot; (ii) secondly *in silico* analysis can generate evidence for further *in vitro* studies especially in relation to metabolic complementarily and signaling and (iii) *in planta* studies involving microscopy and “omic” analysis might then validate the interactions model(s) extrapolated from *in vitro* and *in silico* investigations. For example, initial studies along this line have begun to show via genome sequencing that residents commonly isolated from olive knots possess putative metabolic pathways that are not complete in *Psv* which might lead to complementarity in the metabolism of compounds (e.g., for the mineralizing of L-rhamnose, D-galactose, L-arabinose, SA, and aminoethylphosphonic acid) found in the olive knot.

Many aspects merit further studies such as: (i) the effect of location, olive cultivar, environmental conditions on the relative abundance of the main endophytes inside the knots, (ii) the genetic variability of some endophytes, and (iii) the eventual translocation in the xylem of the endophytic bacteria as well as their fate away from the olive knots.

In conclusion, studies on bacterial plant diseases have thus far historically focused on single species (the pathogen), while little attention has been given on the many other microorganisms most likely present in the infection sites such as soft rotted tissues (Sturz and Matheson, 1996), cankers (Moretti et al., 2007), and tumors (Nautiyal and Dion, 1990). Microbiota living in the infection sites can possibly modulate the severity of the disease, establishing mutual, commensal, and antagonistic interactions as well as interacting with plant defense systems. These potentially complex interactions are largely unknown and in our view merit attention.

In this review, we report the results achieved to date on the multispecies aspect of the olive knot disease. These as well as the hypotheses drawn from the scientific literature in addition to *in silico* analysis indicate that the tumors caused by *Psv* in olive trees represent an ideal niche and an excellent model to study interspecies interactions, sociomicrobiology, evolution, cooperation, and competition of bacteria and could serve as a paradigm in plant pathology. These interactions could result in the evasion of the immune response, chemical signaling, metabolic exchange, and resident-to-pathogen switch (Venturi and Passos da Silva, 2012). The sequencing of all the microbial members as well as the olive plant (http://genomes.cribi.unipd.it/olive/wordpress/welcome/) will be of importance in order to set up future experiments designed to understand interactions. It is likely that these kinds of studies will lead the way for other scientists investigating the potential role of other microbes present in the infection sites of plant diseases. Understanding these interactions could be of crucial importance in designing new and effective strategies for disease control.

### Conflict of Interest Statement

The authors declare that the research was conducted in the absence of any commercial or financial relationships that could be construed as a potential conflict of interest.

## References

[B1] Aragón I. M. Pérez-Martínez I. Moreno-Pérez A. Cerezo M. Ramos C. (2014). New insights into the role of indole-3-acetic acid in the virulence of *Pseudomonas savastanoi* pv. savastanoi. FEMS Microbiol. Lett. 356, 184–192. 10.1111/1574-6968.12413 24606017

[B2] Aragón I. M. Pérez-Mendoza D. Gallegos M. T. Ramos C. (2015). The c-di-GMP phosphodiesterase BifA is involved in the virulence of bacteria from the *Pseudomonas syringae* complex. Mol. Plant Pathol. 10.1111/mpp.12218 25385023 PMC6638514

[B3] Bahar O. Goffer T. Burdman S. (2009). Type IV pili are required for virulence, twitching motility, and biofilm formation of *Acidovorax avenae* subsp citrulli. Mol. Plant Microbe Interact. 22, 909–920. 10.1094/mpmi-22-8-0909 19589067

[B4] Barash I. Manulis-Sasson S. (2009). Recent evolution of bacterial pathogens: the gall-forming *Pantoea agglomerans* case. Annu. Rev. Phytopathol. 47, 133–152. 10.1146/annurev-phyto-080508-081803 19400643

[B5] Bardaji L. Pérez-Martínez I. Rodríguez-Moreno L. Rodríguez-Palenzuela P. Sundin G. W. Ramos C. (2011). Sequence and role in virulence of the three plasmid complement of the model tumor-inducing bacterium *Pseudomonas savastanoi* pv. *savastanoi NCPPB* 3335. PLoS ONE 6:e25705. 10.1371/journal.pone.0025705 22022435 PMC3191145

[B6] Barken K. B. Pamp S. J. Yang L. Gjermansen M. Bertrand J. J. Klausen M. (2008). Roles of type IV pili, flagellum-mediated motility and extracellular DNA in the formation of mature multicellular structures in *Pseudomonas aeruginosa* biofilms. Environ. Microbiol. 10, 2331–2343. 10.1111/j.1462-2920.2008.01658.x 18485000

[B7] Barny M. A. Guinebretiere M. H. Marcais B. Coissac E. Paulin J. P. Laurent J. (1990). Cloning of a large gene-cluster involved in *Erwinia amylovora* CFBP1430 virulence. Mol. Microbiol. 4, 777–786. 10.1111/j.1365-2958.1990.tb00648.x 2117695

[B8] Blango M. G. Mulvey M. A. (2009). Bacterial landlines: contact-dependent signaling in bacterial populations. Curr. Opin. Microbiol. 12, 177–181. 10.1016/j.mib.2009.01.011 19246237 PMC2668724

[B9] Block A. Alfano J. R. (2011). Plant targets for *Pseudomonas syringae* type III effectors: virulence targets or guarded decoys? *Curr*. Opin. Microbiol. 14, 39–46. 10.1016/j.mib.2010.12.011 21227738 PMC3040236

[B10] Boatwright J. L. Pajerowska-Mukhtar K. (2013). Salicylic acid: an old hormone up to new tricks. Mol. Plant Pathol. 14, 623–634. 10.1111/mpp.12035 23621321 PMC6638680

[B11] Borlee B. R. Goldman A. D. Murakami K. Samudrala R. Wozniak D. J. Parsek M. R. (2010). *Pseudomonas aeruginosa* uses a cyclic-di-GMP-regulated adhesin to reinforce the biofilm extracellular matrix. Mol. Microbiol. 75, 827–842. 10.1111/j.1365-2958.2009.06991.x 20088866 PMC2847200

[B12] Boureau T. Elmaarouf-Bouteau H. Garnier A. Brisset M.N. Perino C. Pucheu I. (2006). DspA/E, a type III effector essential for *Erwinia amylovora* pathogenicity and growth in planta, induces cell death in host apple and nonhost tobacco plants. Mol. Plant Microbe Interact. 19, 16–24. 10.1094/mpmi-19-0016 16404949

[B13] Buonaurio R. (2008). “Infection and plant defense responses during plant–bacterial interaction,” in Plant–Microbe Interactions, eds Barka E. A. Clement C. (Kerala, India: Research Signpost), 169–197.

[B14] Burmølle M. Ren D. Bjarnsholt T. Sørensen S. J. (2014). Interactions in multispecies biofilms: do they actually matter? Trends Microbiol. 22, 84–91. 10.1016/j.tim.2013.12.004 24440178

[B15] Burmølle M. Thomsen T. R. Fazli M. Dige I. Christensen L. Homøe P. (2010). Biofilms in chronic infections - a matter of opportunity - monospecies biofilms in multispecies infections. FEMS Immunol. Med. Microbiol. 59, 324–336. 10.1111/j.1574-695X.2010.00714.x 20602635

[B16] Burmølle M. Webb J. S. Rao D. Hansen L. H. Sørensen S. J. Kjelleberg S. (2006). Enhanced biofilm formation and increased resistance to antimicrobial agents and bacterial invasion are caused by synergistic interactions in multispecies biofilms. Appl. Environ. Microbiol. 72, 3916–3923. 10.1128/aem.03022-05 16751497 PMC1489630

[B17] Burr T. J. Katz B. H. Abawi G. S. Crosier D. C. (1991). Comparison of tumorigenic strains of *Erwinia herbicola* isolated from table beet with *Erwima’s h. gypsophilae*. Plant Dis. 75, 855–858.

[B18] Cameron R. K. Zaton K. (2004). Intercellular salicylic acid accumulation is important for age-related resistance in *Arabidopsis* to *Pseudomonas syringae*. Physiol. Mol. Plant Pathol. 65, 197–209. 10.1016/j.pmpp.2005.02.002

[B19] Capasso R. Cristinzio G. Evidente A. Visca C. Iannini C. (1997). “Oleuropein and other polyphenols from olive (*Olea europaea*) for protecting the plant against *Pseudomonas syringae* subsp. *savastanoi*,” in Pseudomonas syringae Pathovars and Related Pathogens, eds Rudolph K. Burr T. J. Mansfield J. W. Stead D. Vivian A. von Kietzell J. (Dordrecht: Kluwer Academic Publishers), 133–137.

[B20] Capasso R. Evidente A. Schivo L. Orru G. Marcialis M. A. Cristinzio G. (1995). Antibacterial polyphenols from olive oil mill waste-waters. J. Appl. Bacteriol. 79, 393–398. 10.1111/j.1365-2672.1995.tb03153.x 7592132

[B21] Cayuela J. A. Rada M. Rios J. J. Albi T. Guinda A. (2006). Changes in phenolic composition induced by *Pseudomonas savastanoi* pv. *savastanoi* infection in olive tree: presence of large amounts of verbascoside in nodules of tuberculosis disease. J. Agr. Food Chem. 54, 5363–5368. 10.1021/jf060807w 16848518

[B22] Chang J. H. Desveaux D. Creason A. L. (2014). The ABCs and 123s of bacterial secretion systems in plant pathogenesis. Annu. Rev. Phytopathol. 52, 317–345. 10.1146/annurev-phyto-011014-015624 24906130

[B23] Compant S. Clement C. Sessitsch A. (2010). Plant growth-promoting bacteria in the rhizo- and endosphere of plants: their role, colonization, mechanisms involved and prospects for utilization. Soil Biol. Biochem. 42, 669–678. 10.1016/j.soilbio.2009.11.024

[B24] Cooksey D. A. (1986). Galls of *Gypsophila paniculata* caused by *Erwinia herbicola*. Plant Dis. 70, 464–468. 10.1094/pd-70-464

[B25] Cortese C. Pérez-Martínez I. Ramos C. Buonaurio R. Moretti C. (2014). “The endophytes *Pantoea agglomerans* and *Erwinia oleae* colocalize with *Pseudomonas savastanoi* pv. *savastanoi* in the olive knots” in XX Convegno Nazionale Società di Patologia Vegetale, Environmentally loyal plant protection: from nano- to field-scale, 31.

[B26] Coutinho B. G. Licastro D. Mendonca-Previato L. Camara M. Venturi V. (2015). Plant-influenced gene expression in the rice endophyte *Burkholderia kururiensis* M130. Mol. Plant Microbe Interact. 28, 10–21. 10.1094/mpmi-07-14-0225-r 25494355

[B27] Coutinho B. G. Mitter B. Talbi C. Sessitsch A. Bedmar E. J. Halliday N. (2013). Regulon studies and in planta role of the BraI/R quorum-sensing system in the plant-beneficial Burkholderia cluster. Appl. Environ. Microbiol. 79, 4421–4432. 10.1128/AEM.00635-13 23686262 PMC3697526

[B28] Danhorn T. Fuqua C. (2007). Biofilm formation by plant-associated bacteria. Annu. Rev. Microbiol. 61, 401–422. 10.1146/annurev.micro.61.080706.093316 17506679

[B29] De Beer D. Stoodley P. (2006). “Microbial biofilms,” in The Prokaryotes, eds Dworkin M. Falkow S. Rosenberg E. Schleifer K. Stackebrandt E. (New York: Springer), 904–937.

[B30] Degrave A. Moreau M. Launay A. Barny M.-A. Brisset M.-N. Patrit O. (2013). The bacterial effector DspA/E is toxic in *Arabidopsis thaliana* and is required for multiplication and survival of fire blight pathogen. Mol. Plant Pathol. 14, 506–517. 10.1111/mpp.12022 23634775 PMC6638835

[B31] Demuyser L. Jabra-Rizk M. A. Van Dijck P. (2014). Microbial cell surface proteins and secreted metabolites involved in multispecies biofilms. Pathog. Dis. 70, 219–230. 10.1111/2049-632x.12123 24376219

[B32] De Vargas Roditi L. Boyle K. E. Xavier J. B. (2013). Multilevel selection analysis of a microbial social trait. Mol. Syst. Biol. 9. 10.1038/msb.2013.42 23959025 PMC3779802

[B33] DeYoung R. M. Copeman R. J. Hunt R. S. (1998). Two strains in the genus *Erwinia* cause galls on douglas-fir in southwestern British Columbia. Can. J. Plant Pathol. 20, 194–200.

[B34] Ercolani G. L. (1971). Presenza epifitica di *Pseudomonas savastanoi* (E. F. Smith) Stevens sull’Olivo, in Puglia. Phytopathol. Mediterr. 10, 130–132.

[B35] Ercolani G. L. (1978). *Pseudomonas savastanoi* and other bacteria colonizing the surface of olive leaves in the field. J. Gen. Microbiol. 109, 245–257.

[B36] Fernandes A. Marcelo M. (2002). “A possible synergistic effect of *Erwinia* sp. on the development of olive knot symptoms caused by *Pseudomonas syringae* pv. savastanoi in *Olea europaea*” in Proceedings of the Fourth International Symposium on Olive Growing, Vols. 1 and 2, eds Vitagliano C. Martelli G. P. (Valenzano: ISHS Acta Horticulturae), 729–731.

[B37] Fuqua C. Parsek M. R. Greenberg E. P. (2001). Regulation of gene expression by cell-to-cell communication: acyl-homoserine lactone quorum sensing. Annu. Rev. Genet. 35, 439–468. 10.1146/annurev.genet.35.102401.090913 11700290

[B38] Gaudriault S. Malandrin L. Paulin J. P. Barny M. A. (1997). DspA, an essential pathogenicity factor of *Erwinia amylovora* showing homology with AvrE of *Pseudomonas syringae*, is secreted via the Hrp secretion pathway in a DspB-dependent way. Mol. Microbiol. 26, 1057–1069. 10.1046/j.1365-2958.1997.6442015.x 9426142

[B39] Gaudriault S. Paulin J. P. Barny M. A. (2002). The DspB/F protein of *Erwinia amylovora* is a type III secretion chaperone ensuring efficient intrabacterial production of the Hrp-secreted DspA/E pathogenicity factor. Mol. Plant Pathol. 3, 313–320. 10.1046/j.1364-3703.2002.00124.x 20569339

[B40] Gavini F. Mergaert J. Beji A. Mielcarek C. Izard D. Kersters K. (1989). Transfer of *Enterobacter agglomerans* (Beijerinck 1888) Ewing and Fife 1972 to Pantoea gen. *nov.* as *Pantoea agglomerans comb. nov*. and description of *Pantoea dispersa* sp. nov. Int. J. Syst. Bacteriol. 39, 337–345.

[B41] Gibbins L. N. (1978). “Erwinia herbicola: a review and perspective,” in Fourth International Conference on Plant Pathogenic Bacteria, Angers, France, 403–431.

[B42] Gomez-Lama Cabanas C. Schilirò E. Valverde-Corredor A. Mercado-Blanco J. (2014). The biocontrol endophytic bacterium *Pseudomonas fluorescens* PICF7 induces systemic defense responses in aerial tissues upon colonization of olive roots. Front. Microbiol. 5:427. 10.3389/fmicb.2014.00427 25250017 PMC4155815

[B43] Gottig N. Garavaglia B. S. Garofalo C. G. Orellano E. G. Ottado J. (2009). A filamentous hemagglutinin-like protein of *Xanthomonas axonopodis* pv. *citri*, the phytopathogen responsible for citrus canker, is involved in bacterial virulence. PLoS ONE 4:e4358. 10.1371/journal.pone.0004358 19194503 PMC2632755

[B44] Guo L. He X. Shi W. (2014). Intercellular communications in multispecies oral microbial communities. Front. Microbiol. 5:328. 10.3389/fmicb.2014.00328 25071741 PMC4076886

[B45] Hachani A. Allsopp L. P. Oduko Y. Filloux A. (2014). The VgrG proteins are “a la Carte” delivery systems for bacterial type VI effectors. J. Biol. Chem. 289, 17872–17884. 10.1074/jbc.M114.563429 24794869 PMC4067218

[B46] Hallmann J. Quadthallmann A. Mahaffee W. F. Kloepper J. W. (1997). Bacterial endophytes in agricultural crops. Can. J. Microbiol. 43, 895–914.

[B47] Hangler M. Burmølle M. Schneider I. Allermann K. Jensen B. (2009). The serine protease esperase HPF inhibits the formation of multispecies biofilm. Biofouling 25, 667–674. 10.1080/08927010903096008 20183125

[B48] Harwood C. S. Parales R. E. (1996). The beta-ketoadipate pathway and the biology of self-identity. Annu. Rev. Microbiol. 50, 553–590. 10.1146/annurev.micro.50.1.553 8905091

[B49] Heindl J. E. Wang Y. Heckel B. C. Mohari B. Feirer N. Fuqua C. (2014). Mechanisms and regulation of surface interactions and biofilm formation in *Agrobacterium*. Front. Plant Sci. 5:176. 10.3389/fpls.2014.00176 24834068 PMC4018554

[B50] Hosni T. (2010). Interaction Between Pseudomonas savastanoi pv. savastanoi, the Causal Agent of Olive Knot, and the Endophytic Bacterial Species Associated With the Knot. Ph.D. thesis. University of Perugia, Perugia, Italy.

[B51] Hosni T. Moretti C. Devescovi G. Suarez-Moreno Z. R. Barek Fatmi M. Guarnaccia C. (2011). Sharing of quorum-sensing signals and role of interspecies communities in a bacterial plant disease. ISME J. 5, 1857–1870. 10.1038/ismej.2011.65 21677694 PMC3223305

[B52] Iacobellis N. S. (2001). “Olive knot,” in Encyclopedia of Plant Pathology, eds Maloy O. C. Murray T. D. (New York: John Wiley and Sons), 713–715.

[B53] Jefferson K. K. (2004). What drives bacteria to produce a biofilm? FEMS Microbiol. Lett. 236, 163–173. 10.1016/j.femsle.2004.06.005 15251193

[B54] Jones J. D. G. Dangl J. L. (2006). The plant immune system. Nature 444, 323–329. 10.1038/nature05286 17108957

[B55] Jousset A. Eisenhauer N. Materne E. Scheu S. (2013). Evolutionary history predicts the stability of cooperation in microbial communities. Nat. Commun. 4, 2573. 10.1038/ncomms3573 24113642

[B56] Kerényi A. Bihary D. Venturi V. Pongor S. (2013). Stability of multispecies bacterial communities: signaling networks may stabilize microbiomes. PLoS ONE 8:e57947. 10.1371/journal.pone.0057947 23483950 PMC3587416

[B57] Kobayashi D. Y. Palumbo J. D. (2000). “Bacterial endophytes and their effects on plants and uses in agriculture,” in Microbial Endophytes, eds Bacon C. W. White J. F. (New York: Marcel Dekker), 199–233.

[B58] Kolenbrander P. E. Palmer R. J. Jr. Periasamy S. Jakubovics N. S. (2010). Oral multispecies biofilm development and the key role of cell–cell distance. Nat. Rev. Microbiol. 8, 471–480. 10.1038/nrmicro2381 20514044

[B59] Koskiniemi S. Lamoureux J. G. Nikolakakis K. C. De Roodenbeke C. T. K. Kaplan M. D. Low D. A. (2013). Rhs proteins from diverse bacteria mediate intercellular competition. Proc. Natl. Acad. Sci. U.S.A. 110, 7032–7037. 10.1073/pnas.1300627110 23572593 PMC3637788

[B60] Lindow S. E. Brandl M. T. (2003). Microbiology of the phyllosphere. Appl. Environ. Microbiol. 69, 1875–1883. 10.1128/aem.69.4.1875-1883.2003 12676659 PMC154815

[B61] Lundberg D. S. Lebeis S. L. Paredes S. H. Yourstone S. Gehring J. Malfatti S. (2012). Defining the core *Arabidopsis thaliana* root microbiome. Nature 488, 86–90. 10.1038/nature11237 22859206 PMC4074413

[B62] Madsen J. S. Burmølle M. Hansen L. H. Sørensen S. J. (2012). The interconnection between biofilm formation and horizontal gene transfer. FEMS Immunol. Med. Microbiol. 65, 183–195. 10.1111/j.1574-695X.2012.00960.x 22444301

[B63] Maldonado-González M. Prieto P. Ramos C. Mercado-Blanco J. (2013). From the root to the stem: interaction between the biocontrol root endophyte *Pseudomonas fluorescens* PICF7 and the pathogen *Pseudomonas savastanoi* NCPPB 3335 in olive knots. Microb. Biotechnol. 6, 275–287. 10.1111/1751-7915.12036 23425069 PMC3815922

[B64] Manulis S. Barash I. (2003). Pantoea agglomerans pvs. gypsophilae and betae, recently evolved pathogens? Mol. Plant Pathol. 4, 307–314. 10.1046/J.1364-3703.2003.00178.X 20569391

[B65] Marchi G. Mori B. Pollacci P. Mencuccini M. Surico G. (2009). Systemic spread of *Pseudomonas savastanoi* pv. *savastanoi* in olive explants. Plant Pathol. 58, 152–158. 10.1111/j.1365-3059.2008.01935.x

[B66] Marchi G. Sisto A. Cimmino A. Andolfi A. Cipriani M. G. Evidente A. (2006). Interaction between *Pseudomonas savastanoi* pv. *savastanoi* and *Pantoea agglomerans* in olive knots. Plant Pathol. 55, 614–624. 10.1111/j.1365-3059.2006.01449.x

[B67] Martínez-García P. M. Ruano-Rosa D. Schiliro E. Prieto P. Ramos C. Rodríguez-Palenzuela P. (2015). Complete genome sequence of *Pseudomonas fluorescens* strain PICF7, an indigenous root endophyte from olive (*Olea europaea* L.) and effective biocontrol agent against *Verticillium dahliae*. Stand. Genomic Sci. 10, 10–10. 10.1186/1944-3277-10-10 25685259 PMC4322347

[B68] Matas I. M. Lambertsen L. Rodríguez-Moreno L. Ramos C. (2012). Identification of novel virulence genes and metabolic pathways required for full fitness of *Pseudomonas savastanoi* pv. *savastanoi* in olive (*Olea europaea*) knots. New Phytol. 196, 1182–1196. 10.1111/j.1469-8137.2012.04357.x 23088618

[B69] Matas I. M. Pilar Castaneda-Ojeda M. Aragon I. M. Antunez-Lamas M. Murillo J. Rodríguez-Palenzuela P. (2014). Translocation and functional analysis of *Pseudomonas savastanoi* pv. *savastanoi* NCPPB 3335 type III secretion system effectors reveals two novel effector families of the *Pseudomonas syringae* complex. Mol. Plant Microbe Interact. 27, 424–436. 10.1094/mpmi-07-13-0206-r 24329173

[B70] Mercado-Blanco J. Rodriguez-Jurado D. Hervas A. Jimenez-Diaz R. M. (2004). Suppression of Verticillium wilt in olive planting stocks by root-associated fluorescent *Pseudomonas* spp. Biol. Control 30, 474–486. 10.1016/j.biocontrol.2004.02.002

[B71] Mitri S. Foster K. R. (2013). The genotypic view of social interactions in microbial communities. Annu. Rev. Genet. 47, 247–273. 10.1146/annurev-genet-111212-133307 24016192

[B72] Mitter B. Petric A. Shin M. W. Chain P. S. G. Hauberg-Lotte L. Reinhold-Hurek B. (2013). Comparative genome analysis of *Burkholderia phytofirmans* PsJN reveals a wide spectrum of endophytic lifestyles based on interaction strategies with host plants. Front. Plant Sci. 4:120. 10.3389/fpls.2013.00120 23641251 PMC3639386

[B73] Moretti C. Cortese C. Passos da Silva D. Devescovi G. Torelli E. Venturi V. (2014a). “Draft genome sequence of a hypersensitive reaction-inducing Pantoea agglomerans strain isolated from olive knots caused by *Pseudomonas savastanoi* pv. *Savastanoi*,” in 13th International Conference on Plant Pathogenic Bacteria (Shanghai, China), S1–P2. 10.1128/genomeA.00774-14 PMC4125774 25103763

[B74] Moretti C. Cortese C. Passos da Silva D. Venturi V. Firrao G. Buonaurio R. (2014b). Draft genome sequence of *Erwinia oleae*, a bacterium associated with olive knots caused by *Pseudomonas savastanoi* pv. *savastanoi*. Genome Announc. 2, e01308–14. 10.1128/genomeA.01308-14 25502684 PMC4263846

[B75] Moretti C. Cortese C. Passos da Silva D. Venturi V. Ramos C. Firrao G. (2014c). Draft genome sequence of *Pseudomonas savastanoi* pv. *savastanoi* strain *DAPP-PG* 722, isolated in Italy from an olive plant affected by knot disease. Genome Announc. 2, e00864–14. 10.1128/genomeA.00864-14 25278521 PMC4183865

[B76] Moretti C. Cortese C. Passos da Silva D. Venturi V. Torelli E. Firrao G. (2014d). Draft genome sequence of a hypersensitive reaction-inducing *Pantoea* agglomerans strain isolated from olive knots caused by *Pseudomonas savastanoi* pv. *savastanoi*. Genome Announc. 2, e00774–14. 10.1128/genomeA.00774-14 25103763 PMC4125774

[B77] Moretti C. Hosni T. Vandemeulebroecke K. Brady C. De Vos P. Buonaurio R. (2011). Erwinia oleae sp nov., isolated from olive knots caused by *Pseudomonas savastanoi pv. savastanoi*. Int. J. Syst. Evol. Microbiol. 61, 2745–2752. 10.1099/ijs.0.026336-0 21186287

[B78] Moretti C. Silvestri F. M. Rossini E. Natalini G. Buonaurio R. (2007). A protocol for rapid identification of *Brenneria nigrifluens* among bacteria isolated from bark cankers in Persian walnut plants. J. Plant Pathol. 89, 211–218.

[B79] Nautiyal C. S. Dion P. (1990). Characterization of the opine-utilizing microflora associated with samples of soil and plants. Appl. Environ. Microbiol. 56, 2576–2579. 16348265 10.1128/aem.56.8.2576-2579.1990 PMC184770

[B80] Oh C.-S. Martin G. B. Beer S. V. (2007). DspA/E, a type III effector of Erwinia amylovora, is required for early rapid growth in *Nicotiana benthamiana* and causes NbSGT1-dependent cell death. Mol. Plant Pathol. 8, 255–265. 10.1111/j.1364-3703.2007.00390.X 20507497

[B81] Opgenorth D. C. Takikawa Y. Hendson M. Clark E. (1994). First report of bacterial gall of *Wisteria sinensis* caused by *Erwinia herbicola* pv *milletiae* in California. Plant Dis. 78, 1217–1217.

[B82] Ouzari H. Khsairi A. Raddadi N. Jaoua L. Hassen A. Zarrouk M. (2008). Diversity of auxin-producing bacteria associated to *Pseudomonas savastanoi*-induced olive knots. J. Basic Microb. 48, 370–377. 10.1002/jobm.200800036 18759227

[B83] Pardo F. Perich F. Villarroel L. Torres R. (1993). Isolation of verbascoside, an antimicrobial constituent of *Buddleja globosa* leaves. J. Ethnopharmacol. 39, 221–222. 8258981 10.1016/0378-8741(93)90041-3

[B84] Parsek M. R. Greenberg E. P. (2005). Sociomicrobiology: the connections between quorum sensing and biofilms. Trends Microbiol. 13, 27–33. 10.1016/j.tim.2004.11.007 15639629

[B85] Passos da Silva D. Devescovi G. Paszkiewicz K. Moretti C. Buonaurio R. Studholme D. J. (2013). Draft genome sequence of *Erwinia toletana*, a bacterium associated with olive knots caused by *Pseudomonas savastanoi* pv. savastanoi. Genome Announc. 1, e00205–13. 10.1128/genomeA.00205-13 23661482 PMC3650441

[B86] Passos da Silva D. Pilar Castaneda-Ojeda M. Moretti C. Buonaurio R. Ramos C. Venturi V. (2014). Bacterial multispecies studies and microbiome analysis of a plant disease. Microbiology 160, 556–566. 10.1099/mic.0.074468-0 24421406

[B87] Penyalver R. Garcia A. Ferrer A. Bertolini E. Quesada J. M. Salcedo C. I. (2006). Factors affecting *Pseudomonas savastanoi* pv. *savastanoi* plant inoculations and their use for evaluation of olive cultivar susceptibility. Phytopathology 96, 313–319. 10.1094/phyto-96-0313 18944447

[B88] Pérez-Martínez I. Rodríguez-Moreno L. Lambertsen L. Matas I. M. Murillo J. Tegli S. (2010). Fate of a *Pseudomonas savastanoi* pv. *savastanoi* type III secretion system mutant in olive plants (*Olea europaea L.*). Appl. Environ. Microbiol. 76, 3611–3619. 10.1128/aem.00133-10 20363790 PMC2876471

[B89] Pérez-Mendoza D. Aragon I. M. Prada-Ramirez H. A. Romero-Jimenez L. Ramos C. (2014). Responses to elevated c-di-GMP levels in mutualistic and pathogenic plant-interacting bacteria. PLoS ONE 9:e91645. 10.1371/journal.pone.0091645 24626229 PMC3953490

[B90] Petri L. (1907). Untersuchungen über die Identität des Rotzbacillus des Oelbaumes. Zentrallblatt für Bakteriologie, Parasitenkunde, Infektionskrankheiten und Hygiene XIX, 531–538.

[B91] Prieto P. Navarro-Raya C. Valverde-Corredor A. Amyotte S. G. Dobinson K. F. Mercado-Blanco J. (2009). Colonization process of olive tissues by *Verticillium dahliae* and its in planta interaction with the biocontrol root endophyte *Pseudomonas fluorescens* PICF7. Microb. Biotechnol. 2, 499–511. 10.1111/j.1751-7915.2009.00105.x 21255281 PMC3815910

[B92] Quesada J. M. Garcia A. Bertolini E. Lopez M. M. Penyalver R. (2007). Recovery of *Pseudomonas savastanoi* pv. *savastanoi* from symptomless shoots of naturally infected olive trees. Int. Microbiol. 10, 77–84. 10.2436/20.1501.01.11 17661284

[B93] Quesada J. M. Penyalver R. López M. M. (2012). “Epidemiology and control of plant diseases caused by phytopathogenic bacteria: the case of olive knot disease caused by *Pseudomonas savastanoi* pv. *savastanoi*,” in Plant Pathology, ed. Cumagun C. J. (INTECH Open Access Publisher), 299–326.

[B94] Quesada J. M. Penyalver R. Pérez-Panades J. Salcedo C. I. Carbonell E. A. Lopez M. M. (2010). Dissemination of *Pseudomonas savastanoi* pv. *savastanoi* populations and subsequent appearance of olive knot disease. Plant Pathol. 59, 262–269. 10.1111/j.1365-3059.2009.02200.x

[B95] Ramos C. Matas I. M. Bardaji L. Aragon I. M. Murillo J. (2012). *Pseudomonas savastanoi* pv. *savastanoi*: some like it knot. Mol. Plant Pathol. 13, 998–1009. 10.1111/j.1364-3703.2012.00816.x 22805238 PMC6638699

[B96] Ramsey M. M. Rumbaugh K. P. Whiteley M. (2011). Metabolite cross-feeding enhances virulence in a model polymicrobial infection. PLoS Pathog. 7:e1002012. 10.1371/journal.ppat.1002012 21483753 PMC3069116

[B97] Reinhold-Hurek B. Hurek T. (2011). Living inside plants: bacterial endophytes. Curr. Opin. Plant Biol. 14, 435–443. 10.1016/j.pbi.2011.04.004 21536480

[B98] Rodríguez-Moreno L. Barcelo-Munoz A. Ramos C. (2008). *In vitro* analysis of the interaction of *Pseudomonas savastanoi* pvs. *savastanoi* and *nerii* with micropropagated olive plants. Phytopathology 98, 815–822. 10.1094/phyto-98-7-0815 18943258

[B99] Rodríguez-Moreno L. Jimenez A. J. Ramos C. (2009). Endopathogenic lifestyle of *Pseudomonas savastanoi* pv. *savastanoi* in olive knots. Microb. Biotechnol. 2, 476–488. 10.1111/j.1751-7915.2009.00101.x 21255279 PMC3815908

[B100] Rodríguez-Palenzuela P. Matas I. M. Murillo J. Lopez-Solanilla E. Bardaji L. Pérez-Martínez I. (2010). Annotation and overview of the *Pseudomonas savastanoi* pv. *savastanoi NCPPB* 3335 draft genome reveals the virulence gene complement of a tumour-inducing pathogen of woody hosts. Environ. Microbiol. 12, 1604–1620. 10.1111/j.1462-2920.2010.02207.x 20370821

[B101] Rojas A. M. de los Rios J. E. G. D. L. Saux M. F.-L. Jimenez P. Reche P. Bonneau S. (2004). Erwinia toletana sp. *nov*., associated with *Pseudomonas savastanoi*-induced tree knots. Int. J. Syst. Evol. Microbiol. 54, 2217–2222. 10.1099/ijs.0.02924-0 15545461

[B102] Roussos P. A. Pontikis C. A. Tsantili E. (2002). Root promoting compounds detected in olive knot extract in high quantities as a response to infection by the bacterium *Pseudomonas savastanoi* pv. savastanoi. Plant Sci. 163, 533–541. 10.1016/S0168-9452(02)00157-7

[B103] Russell A. B. Peterson S. B. Mougous J. D. (2014). Type VI secretion system effectors: poisons with a purpose. Nat. Rev. Microbiol. 12, 137–148. 10.1038/nrmicro3185 24384601 PMC4256078

[B104] Ryan R. P. Germaine K. Franks A. Ryan D. J. Dowling D. N. (2008). Bacterial endophytes: recent developments and applications. FEMS Microbiol. Lett. 278, 1–9. 10.1111/j.1574-6968.2007.00918.x 18034833

[B105] Savastano L. (1886). Les maladies de l’olivier, et la tuberculose en particulier. *C. R. Séance*. Acad. Agric. Fr. 103, 1144.

[B106] Schilirò E. Ferrara M. Nigro F. Mercado-Blanco J. (2012). Genetic responses induced in olive roots upon colonization by the biocontrol endophytic bacterium *Pseudomonas fluorescens* PICF7. PLoS ONE 7:e48646. 10.1371/journal.pone.0048646 23144916 PMC3492495

[B107] Schwering M. Song J. Louie M. Turner R. J. Ceri H. (2013). Multi-species biofilms defined from drinking water microorganisms provide increased protection against chlorine disinfection. Biofouling 29, 917–928. 10.1080/08927014.2013.816298 23879183

[B108] Sessitsch A. Hardoim P. Doering J. Weilharter A. Krause A. Woyke T. (2012). Functional characteristics of an endophyte community colonizing rice roots as revealed by metagenomic analysis. Mol. Plant Microbe Interact. 25, 28–36. 10.1094/mpmi-08-11-0204 21970692

[B109] Sheng J. S. Citovsky V. (1996). Agrobacterium plant cell DNA transport: have virulence proteins, will travel. Plant Cell 8, 1699–1710. 8914322 10.1105/tpc.8.10.1699 PMC161308

[B110] Sisto A. Cipriani M. G. Morea M. (2004). Knot formation caused by *Pseudomonas syringae* subsp. *savastanoi* on olive plants is hrp-dependent. Phytopathology 94, 484–489. 10.1094/phyto.2004.94.5.484 18943767

[B111] Stewart P. S. (2002). Mechanisms of antibiotic resistance in bacterial biofilms. Int. J. Med. Microbiol. 292, 107–113. 10.1078/1438-4221-00196 12195733

[B112] Sturz A. V. Matheson B. G. (1996). Populations of endophytic bacteria which influence host-resistance to *Erwinia*-induced bacterial soft rot in potato tubers. Plant Soil 184, 265–271. 10.1007/bf00010455

[B113] Subramoni S. Venturi V. (2009). LuxR-family ‘solos’: bachelor sensors/regulators of signalling molecules. Microbiology 155, 1377–1385. 10.1099/mic.0.026849-0 19383698

[B114] Surico G. (1993). Scanning electron microscopy of olive and oleander leaves colonized by *Pseudomonas syringae* subsp. savastanoi. J. Phytopathol. 138, 31–40.

[B115] Surico G. Iacobellis N. S. (1992). “Phytohormones and olive knot disease,” in Molecular Signals in Plant–Microbe Communications, ed. Verma D. P. (Boca Raton, FL: CRC Press), 209–227.

[B116] Surico G. Lavermicocca P. (1989). A semiselective medium for the isolation of *Pseudomonas syringae* pv. savastanoi. Phytopathology 79, 185–190. 10.1094/phyto-79-185

[B117] Tashiro Y. Yawata Y. Toyofuku M. Uchiyama H. Nomura N. (2013). Interspecies interaction between *Pseudomonas aeruginosa* and other microorganisms. Microbes Environ. 28, 13–24. 10.1264/jsme2.ME12167 23363620 PMC4070684

[B118] Temsah M. Hanna L. Saad A. T. (2008). Anatomical pathogenesis of *Pseudomonas savastanoi* on olive and genesis of knots. J. Plant Pathol. 90, 225–232.

[B119] Uccella N. (2001). Olive biophenols: biomolecular characterization, distribution and phytoalexin histochemical localization in the drupes. Trends Food Sci. Technol. 11, 315–327. 10.1016/s0924-2244(01)00029-2

[B120] Ude S. Arnold D. L. Moon C. D. Timms-Wilson T. Spiers A. J. (2006). Biofilm formation and cellulose expression among diverse environmental *Pseudomonas* isolates. Environ. Microbiol. 8, 1997–2011. 10.1111/j.1462-2920.2006.01080.x 17014498

[B121] Varvaro L. Surico G. (1978). Comportamento di diverse cultivars di olivo *Olea europaea* L. alla inoculazione artificiale con Pseudomonas savastanoi E. F. Smith Stevens. Phytopathol. Mediterr. 17, 174–178.

[B122] Vasanthakumar A. McManus P. S. (2004). Indole-3-acetic acid-producing bacteria are associated with cranberry stem gall. Phytopathology 94, 1164–1171. 10.1094/phyto.2004.94.11.1164 18944451

[B123] Venturi V. Fuqua C. (2013). Chemical signaling between plants and plant-pathogenic bacteria. Annu. Rev. Phytopathol. 51, 17–37. 10.1146/annurev-phyto-082712-102239 23915131

[B124] Venturi V. Passos da Silva D. (2012). Incoming pathogens team up with harmless ‘resident’ bacteria. Trends Microbiol. 20, 160–164. 10.1016/j.tim.2012.02.003 22390987

[B125] Wang S. Parsek M. R. Wozniak D. J. Ma L. Z. (2013). A spider web strategy of type IV pili-mediated migration to build a fibre-like Psl polysaccharide matrix in *Pseudomonas aeruginosa* biofilms. Environ. Microbiol. 15, 2238–2253. 10.1111/1462-2920.12095 23425591 PMC4466117

[B126] West S. A. Griffin A. S. Gardner A. Diggle S. P. (2006). Social evolution theory for microorganisms. Nat. Rev. Microbiol. 4, 597–607. 10.1038/nrmicro1461 16845430

[B127] Yaron S. Römling U. (2014). Biofilm formation by enteric pathogens and its role in plant colonization and persistence. Microb. Biotechnol. 7, 496–516. 10.1111/1751-7915.12186 25351039 PMC4265070

[B128] Young J. M. (2004). Olive knot and its pathogens. Australas. Plant Pathol. 33, 33–39. 10.1071/AP03074

